# Meta-analysis of the efficacy of ω-3 polyunsaturated fatty acids when treating patients with polycystic ovary syndrome

**DOI:** 10.1097/MD.0000000000035403

**Published:** 2023-09-29

**Authors:** Yue Huang, Xiang Zhang

**Affiliations:** a Department of Reproductive Medicine, Cangzhou Central Hospital, Cangzhou, Hebei Province, China; b Gynaecology Department Ward 2, Cangzhou Central Hospital, Cangzhou, Hebei Province, China.

**Keywords:** ω-3 polyunsaturated fatty acids, efficacy, meta-analysis, PCOS, polycystic ovary syndrome

## Abstract

**Objective:**

To systematically assess the efficacy of ω-3 polyunsaturated fatty acids (PUFAs) when treating polycystic ovary syndrome (PCOS).

**Methods:**

This meta-analysis follows Preferred Reporting Items for Systematic Reviews and Meta-Analyses criteria. We searched PubMed, EMBASE, ScienceDirect, Cochrane Library, China journal full-text database, VIP full-text Database, Wanfang Database, and Chinese Biomedical Literature Data for clinical trials on ω-3 PUFAs’ efficacy in treating PCOS. Two independent reviewers examined and analyzed studies, resolving inconsistencies through discussion. RevMan5.3 software performed heterogeneity-based fixed and random-effects meta-analysis. We assessed bias using the Cochrane bias risk assessment tool.

**Results:**

Our meta-analysis included 7 clinical control studies comprising 574 samples to evaluate the impact of ω-3 PUFAs on various metabolic markers in PCOS patients. We observed a significant reduction in total cholesterol (TC) and triglyceride (TG) levels (*P* < .05), along with a decrease in insulin resistance as measured by the Homeostatic Model Assessment for Insulin Resistance (HOMA-IR) (*P* < .05). Testosterone (T) levels were also lowered in the study group post-treatment (*P* < .05). However, no notable effects were found on body mass index (BMI), fasting blood sugar (FBS), low-density lipoprotein cholesterol (LDL-C), high-density lipoprotein cholesterol (HDL-C), and Ferriman-Gallwey (mFG) scores (*P* > .05). Publication bias was not detected, enhancing the robustness of our results. Our study suggests that ω-3 PUFAs could be beneficial in managing specific metabolic markers in PCOS, although the results showed marked heterogeneity.

**Conclusion:**

In PCOS patients, PUFAs can enhance reproductive endocrine, glucose, and lipid levels. However, additional research and extended follow-up are required to confirm this.

## 1. Introduction

Polycystic ovary syndrome (PCOS) affects multiple systems of the human body, and has a certain impact on the development of diabetes, stroke, coronary heart disease and other diseases.^[1]^ According to statistics, the prevalence of PCOS among Chinese women aged 19 to 45 is as high as 5.6%.^[2]^ Most PCOS patients present with menstrual disorders, hyperandrogenism, obesity, and insulin resistance.^[3,4]^ The Rotterdam diagnostic criteria stipulates that any 2 of the following requirements must be met for the diagnosis of PCOS: oligogenesis or anovulation; clinical and/or biochemical detection of hyperandrogenism; color Doppler ultrasound showing ovarian polycystic kind of change. In addition, other endocrine diseases similar to PCOS and ovulation disorders caused by other reasons are excluded.^[5]^ With the deepening of the research on the long-term complications of PCOS, a large number of research results have shown that PCOS is one of the risk factors for diabetes.^[6]^ Diabetes and impaired glucose tolerance (IGT) are remarkably more common in PCOS patients than in other women.^[7]^ Moreover, PCOS patients are at 6 times the risk of developing diabetes; they are at a 2 to 5 times greater risk of developing diabetes than the general population.^[8]^ Diabetes-related expenses account for more than 40% of the therapeutic expenses of PCOS patients. Symptoms of PCOS include insulin resistance and hyperinsulinemia, both of which increase the risk of developing diabetes and insulin resistance. Therefore, the management focus of PCOS patients should not be limited to reproductive needs, but also need to pay attention to the risk of long-term diabetes and preventive measures. It is possible to prevent or delay the occurrence and development of diabetes through early detection and lifestyle interventions as well as with drug treatments.

Lifestyle intervention is the first choice of PCOS management, but its treatment duration is poor. Recently, drug therapy has become another effective choice for patients with PCOS. A series of clinical studies have been carried out on the intervention of drugs on the risk of diabetes in patients with PCOS, including metformin, thiazolidinediones, glucagon-like peptide-1 receptor agonists, dipeptidyl peptidase-4 inhibitors, sodium-glucose cotransport protein-2 inhibitors, orlistat and so on. Metformin has still been the first choice for diabetes risk intervention in patients with PCOS. However, some patients still need to actively look for a safer and more effective drug because of gastrointestinal adverse reactions.

Early studies have shown that intake of an appropriate amount of unsaturated fatty acids in the daily diet can remarkably improve dyslipidemia, impaired vascular endothelial function and insulin resistance in PCOS patients,^[9]^ in the Chinese Guidelines to Diagnose and Treat PCOS,^[10]^ unsaturated fatty acids are replaced by saturated fatty acids as one of the methods of dietary intervention. Unsaturated fatty acids (PUFAs) are long-chain fatty acids with 2 or more unsaturated double bond structures and carbon atoms of 18 to 22,^[11]^ which can usually be divided into ω-3 and ω-6 polyunsaturated fatty acids (PUFAs). The double bond furthest from the carboxyl end of a saturated fatty acid is the ω-3 on the penultimate carbon atom, while the ω-6 on the sixth carbon atom.^[12]^ There are unique biological properties associated with omega-3s and omega-6s of the polyunsaturated fatty acid family. They cannot be synthesized by humans and other mammals themselves and must be absorbed from food to meet their nutritional needs, hence they are also known as essential fatty acids. PUFAs are also widely used in the field of biomedicine due to their pharmacological effects. Some studies have shown that PUFAs have anti-platelet aggregation and anti-thrombotic effects, which have been used in cardiovascular disease research.^[13]^ The results of recent investigations have also demonstrated that omega-3 PUFAs have the beneficial effects on lowering blood pressure and can be used in the manufacture and preparation of anti-hypertensive drugs. Epidemiological studies revealed that in the patients with HOMA-insulin resistance, the level of ω-3 PUFAs in serum was remarkably lower than that of normal-weight subjects, which was correlated with body mass index (BMI) negatively.^[14]^

Nowadays, many randomized controlled trials have evaluated the effects of oral supplementation of ω-3 PUFAs on metabolic and endocrine indexes in patients with PCOS, suggesting that ω-3 PUFAs has high application value in the clinical treatment of PCOS patients.^[15]^ Supplementation with natural molecules such as inositols, resveratrol, flavonoids and flavones, vitamin C, vitamin E and vitamin D, and omega-3 fatty acids may contribute to overcoming PCOS pathological features, including the presence of immature oocyte, IR, hyperandrogenism, oxidative stress and inflammation.^[16]^ In addition, Therefore, this study provides a systematic, quantitative, and comprehensive analysis of the results of similar independent studies through a meta-analysis. The effects of oral supplementation of ω-3 PUFAs on metabolic and endocrine indexes in patients with PCOS were evaluated to provide an objective basis for their clinical application and further research. The study provides a systematic, quantitative, and comprehensive analysis of the results of similar independent studies through Meta-analysis to evaluate the effects of oral supplementation of ω-3 PUFAs on metabolic and endocrine indexes in patients with PCOS, providing an objective basis for their clinical application and further research. In this context, it is necessary to demonstrate the therapeutic effects of ω-3 PUFAs on PCOS patients through more and more authoritative scientific studies to offer a theoretical basis to promote and apply this therapy.

## 2. Methods

### 2.1. The sources and retrieval methods of documents

This study adheres to the Preferred Reporting Items for Systematic Reviews and Meta-Analyses guidelines for conducting and reporting meta-analyses. PubMed, EMBASE, ScienceDirect, Cochrane Library, China National Knowledge Infrastructure, VIP full-text Database, Wanfang Database, and Chinese Biomedical Literature data were searched to compile a comprehensive list of articles. We included relevant Chinese and foreign periodicals, conference papers, and degree papers that were focused on the treatment of PCOS with ω-3 PUFAs. The literature search was conducted using both Medical Subject Headings terms and free words (non-medical subject headings terms) in combination with subject words. The key words for the search included “ω-3 polyunsaturated fatty acids,” “PCOS,” “curative effect analysis,” “Efficacy analysis,” and “meta-analysis.” The search range covered literature published from January 2010 to April 2022.

### 2.2. Literature inclusion criteria and exclusion criteria

To ensure a comprehensive understanding of the research landscape, our literature search covered a broad range of studies including relevant Chinese and foreign periodicals, conference papers, and degree papers. However, for the purpose of this meta-analysis, we focused exclusively on clinical controlled trials.

#### 2.2.1. Literature inclusion criteria.

Research types: all the clinical controlled trials of ω-3 PUFAs when treating patients with PCOS; Research objects: According to the European Society for Human Reproduction and Embryology and the American Society for Reproductive Medicine in 2003,^[16]^ PCOS should meet the diagnostic criteria. The diagnosis can be made if 2 of the following 3 items were present. For PCOS: Occasional ovulation and/or anovulation; Clinical and/or biochemical indicators suggest hyperandrogenism; Polycystic ovaries: Ultrasonography shows at least 1 ovarian diameter of 2 ~ 9 mm, ≥12 follicles, and/or increased ovarian volume >10 mL. However, other possible pathogenic factors should be excluded, like congenital adrenocortical hyperplasia, secretory hormone tumor, Cushing syndrome and so on. Intervention: the study group was treated with ω-3 PUFAs, while the control group only received placebo, placebo combined with metformin, or ω-3, which could not influence the result of trials.

#### 2.2.2. Literature exclusion standard.

There was no case-control study conducted. It is impossible to use the data because the report was incomplete. Research content that is repeated will be taken from the most recent studies. No remarkable results were found in the evaluation of the study curative effect. The related literatures were reviewed.

### 2.3. Literature screening process

#### 2.3.1. Primary screening.

During the primary screening stage, we first reviewed the title and abstract of each article obtained through the initial literature search. Articles were excluded at this stage if they clearly did not meet the inclusion criteria or were not relevant to the study question.

#### 2.3.2. Secondary screening.

Articles that passed the primary screening were subjected to a full-text review. In this stage, we applied the inclusion and exclusion criteria in more detail. Two independent reviewers were involved in the assessment, and discrepancies between them were resolved through discussion. Any discrepancies between reviewers at either screening stage were noted and discussed until a consensus was reached. If the paper passed the secondary screening, it was included in the meta-analysis.

### 2.4. Quality evaluation and data extraction

#### 2.4.1. Bias risk assessment contained in the study.

For the evaluation of bias risk, the Cochrane System Review Manual 5.3 bias risk assessment tool was used. Two independent researchers performed the risk assessment, and discrepancies were resolved through discussion.

#### 2.4.2. Literature screening and data extraction.

Literature selection was conducted independently by 2 researchers. Data were extracted and quality was evaluated by these same 2 independent researchers. Any discrepancies were resolved through discussion. Data management and extraction were performed using Note Express and Excel Office software. Data in the literature could be supplemented by the authors of this paper if they were incomplete. The content of data extraction contained: Basic Information: Author, publication time, number of cases. Intervention Measures: Scheme, and course of treatment. Outcome Indicators: Sex hormones, blood glucose, blood lipids, insulin, and other indicators.

### 2.5. Statistical processing

Meta analysis was carried out using RevMan5.3 software. Counting data was indexed by relative risk and measurement data was indexed by mean difference. In addition to the point estimate, the 95% confidence interval (CI) for each effect was provided. χ^2^ test was adopted for heterogeneity test, and I^2^ was adopted to judge the heterogeneity. There are 2 popular statistical models for meta-analysis, the fixed-effect model, and the random-effects model. The fact that these 2 models employ similar sets of formulas to compute statistics, and sometimes yield similar estimates for the various parameters, may lead people to believe that the models are interchangeable. In fact, though, the models represent fundamentally different assumptions about the data. Fixed effect models are used if there is no heterogeneity; if there exhibits heterogeneity, subgroup, sensitivity, or descriptive analysis are adopted, and the random effect model is adopted if there exhibits heterogeneity. The difference exhibited statistically remarkable (*P* < .05). The inverted funnel chart was further drawn to measure the publication bias contained in the literature. Eggers test was used to check the asymmetry of the funnel chart. Whenever the *P* value of this test was <.1, the TrimandFill method could be used to correct the funnel chart and adjust the effect of the potential release deviation.

## 3. Results

### 3.1. Receipt of literature and basic situation of literature inclusion

**Figure 1.**
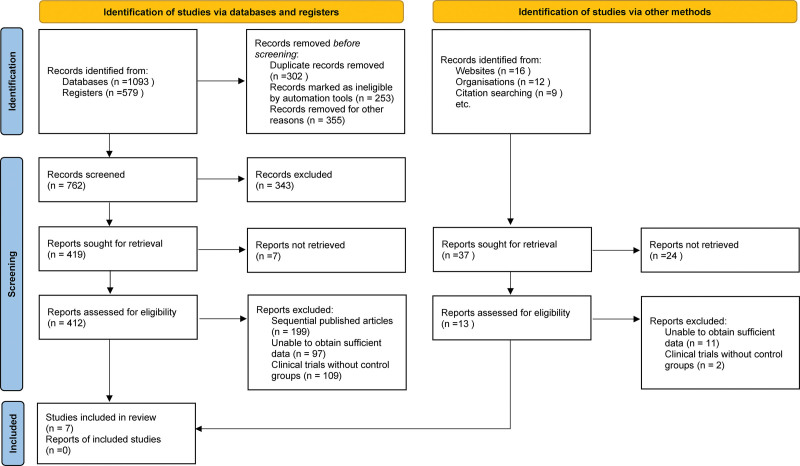
Literature screening diagram.

Initially, our comprehensive search strategy yielded a total of 1672. After removing duplicate records removed, records marked as ineligible by automation tools and records removed for other reasons,762 remained for consideration. During the primary screening stage, we reviewed the titles and abstracts, excluding 343 that did not meet our inclusion criteria. Following a full-text review in the secondary screening stage, 7 clinical control studies ultimately met our criteria and were included in the meta-analysis, comprising a total of 574 samples.^[5,17–22]^ Figure 1 provides a detailed flowchart illustrating the screening and selection process, and Table 1 outlines the basic characteristics of the included literature.

**Table 1:** Basic characteristics of literature.

Include the literature	Yr of publication	N (C/T)	Intervention method		Outcome index	Age (yr)		Course of treatment
			C	T		C	T	
Karakas E^[5]^	2016	17/34	Placebo	ω-3 PUFAs	①②③④⑤⑥⑦	27.73 ± 4.53	27.33 ± 4.27	8 wk
Rahmani E^[17]^	2017	34/34	Placebo + metformin	ω-3 PUFAs + Vitamin C + Metformin	①②③⑥⑦	26.6 ± 5.6	24.9 ± 5.5	12 wk
Mirmasoumi G^[18]^	2018	30/30	Placebo + metformin	ω-3 PUFAs + Metformin	①②③⑤⑥⑦⑧⑨	27.0 ± 3.2	28.4 ± 6.4	12 wk
Khani B^[19]^	2017	44/43	Placebo	ω-3 PUFAs	①②③④⑥⑦⑨	29.23 ± 6.73	31.04 ± 5.04	24 wk
Ebrahimi FA^[20]^	2017	34/34	Placebo	ω-3 PUFAs + Vitamin	①④⑤⑧⑨	25.2 ± 5.2	23.8 ± 4.6	12 wk
Jamilian M^[21]^	2018	20/20	Placebo	ω-3 PUFAs + Vitamin	④⑨	24.4 ± 4.7	22.3 ± 4.7	12 wk
Zhou Yanni^[22]^	2020	100/100	Omega-3	ω-3 PUFAs	①③④⑤⑧	35.51 ± 6.82	35.50 ± 6.81	12 wk

C: control group; T: research group. Omega-3 PUFAs: ω-3 polyunsaturated fatty acids;①Body mass index(BMI);②Total cholesterol (TC);③Triglyceride (TG);④Insulin resistance index (HOMA-IR);⑤Fasting blood glucose (FBS);⑥Low density lipoprotein cholesterol (LDL-C);⑦High density lipoprotein cholesterol (HDL-C);⑧Total testosterone (T);⑨Ferriman-gallery score.

### 3.2. Evaluation of the quality of the methodology contained in the literature

**Figure 2.**
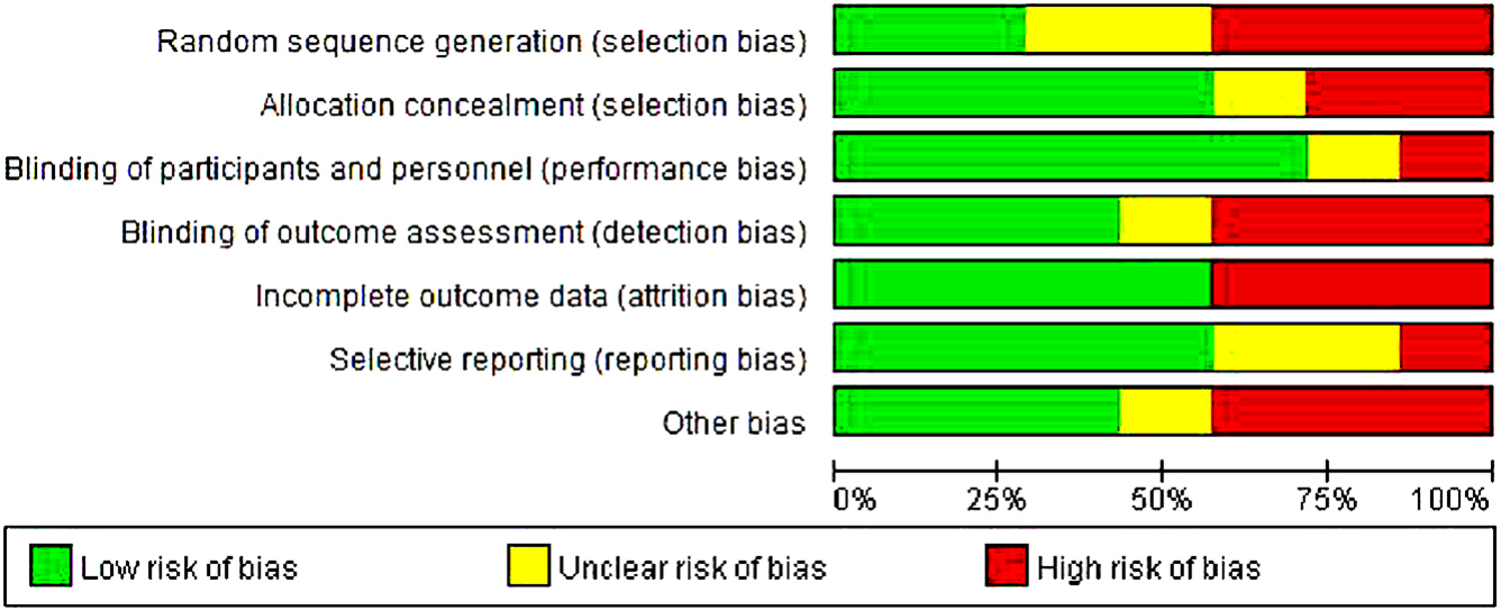
Assessment of risk of bias in this meta-analysis. Risk of bias graph about each risk of bias item presented as percentages across all included studies. The green bar represents low risk of bias, and the yellow bar represents unclear risk of bias; the red bar represents high risk of bias.

**Figure 3.**
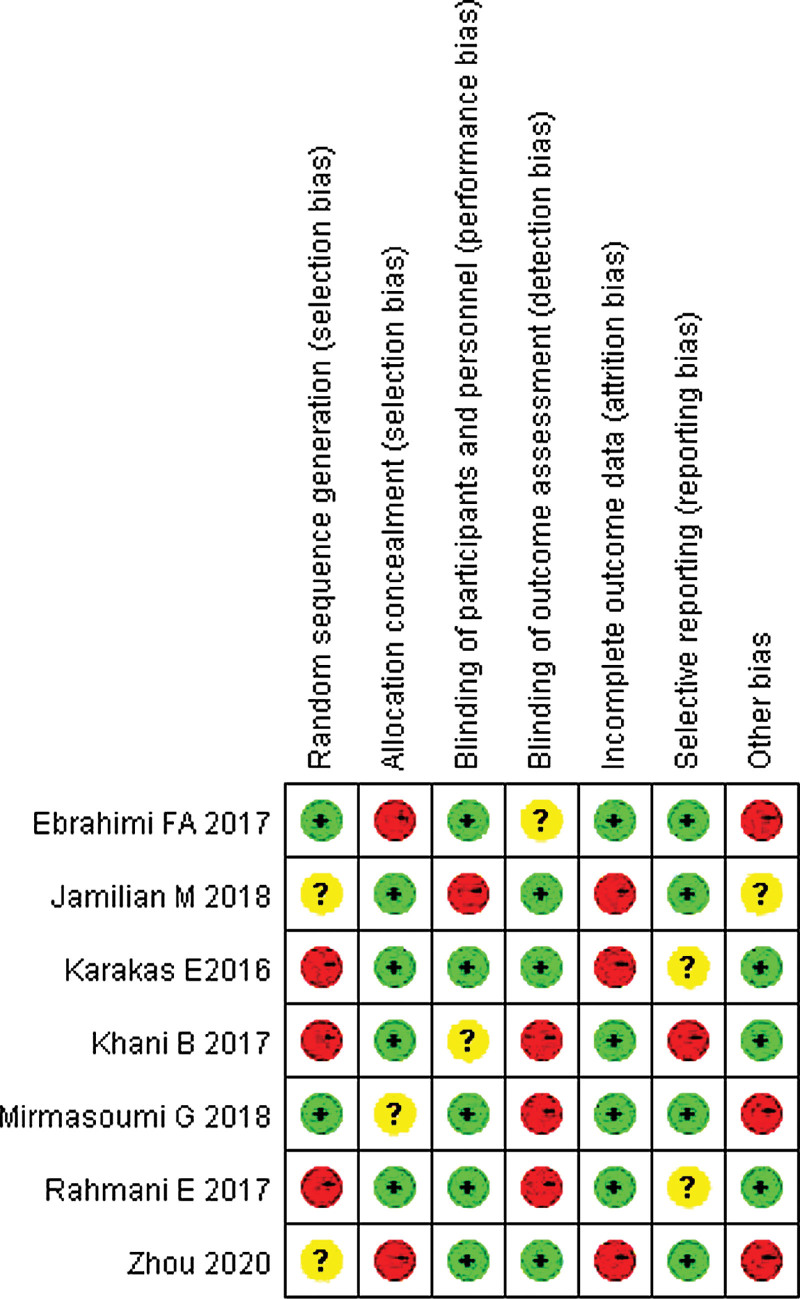
Summary chart of risk bias for each study assessed.

Only part of the literature described the random method in detail, so it is defined as high risk. All the literature did not mention whether the allocation was hidden or not, so it was defined as risk uncertainty. The blind method was not mentioned in all the literature, so the performance risk was defined as high risk and the detection risk was defined as low risk. All the literature reports were complete, there was no missing case data, which was a low risk. None of the literature had obtained its trial plan, so it was classified as risk uncertainty. Some literature reports found other risks, which were high risk. The risk bias analysis is shown in Figures 2 and 3.

### 3.3. Meta analysis result

#### 3.3.1. BMI assessment.

**Figure 4.**
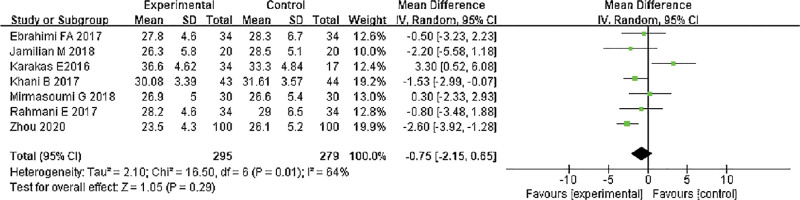
Forest analysis diagram for BMI comparison. BMI = body mass index.

There were 7 clinical controlled studies contained in this study, with 574 samples. The BMI after treatment was analyzed. The results of the heterogeneity test indicated that Chi^2^ = 16.50, df = 6, *P* = .01, I^2^ = 64%, indicating that the research data contained in the study showed distinct heterogeneity. The random effect model was used to analyze (Fig. 4). There were no remarkable differences in BMI after treatment (*P* > .05). It indicated that ω-3 PUFAs had no remarkable effect on the BMI value of PCOS patients. The heterogeneity test indicated that Chi^2^ = 16.50, df = 6, *P* = .01, I^2^ = 64%. The random effect model was used to analyze (*P* > .05).

#### 3.3.2. Total cholesterol level (total cholesterol, TC).

**Figure 5.**
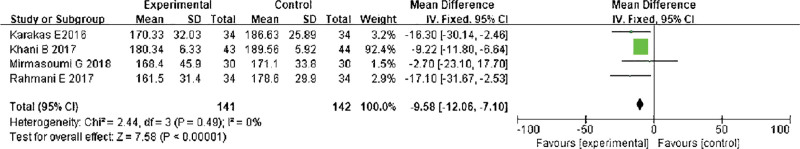
Forest analysis diagram of TC comparison. TC = total cholesterol.

There were 7 clinical controlled studies with 574 samples contained in this study. Meta-analysis was performed on the TC levels. The results of the heterogeneity test indicated that Chi^2^ = 1.97, df = 3, *P* = .58, I^2^ = 0%, indicating that the research data contained in the study showed distinct heterogeneity. The fixed effect model was adopted to analyze (Fig. 5). Following treatment, there exhibited a remarkable reduction in TC levels in the study group (*P* < .05). The heterogeneity test indicated that Chi^2^ = 1.97, df = 3, *P* = .58, I^2^ = 0%. The fixed effect model was adopted to analyze (*P* < .05).

#### 3.3.3. Triglyceride (TG).

**Figure 6.**
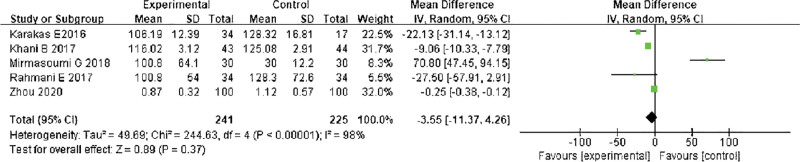
Forest analysis diagram of TG comparison. TG = triglyceride.

The meta-analysis of TG levels after treatment was performed. The results of the heterogeneity test indicated that Chi^2^ = 243.84, df = 4, *P* < .00001, I^2^ = 98%, indicating that the research data contained in the study showed distinct heterogeneity. The random effect model was adopted to analyze (Fig. 6). After treatment, TG levels in the study group were lower in comparison with control group (*P* < .05). The heterogeneity test indicated that Chi^2^ = 243.84, df = 4, *P* < .00001, I^2^ = 98%. The random effect model was adopted to analyze (*P* < .05).

#### 3.3.4. HOMA-insulin resistance (HOMA-IR).

**Figure 7.**
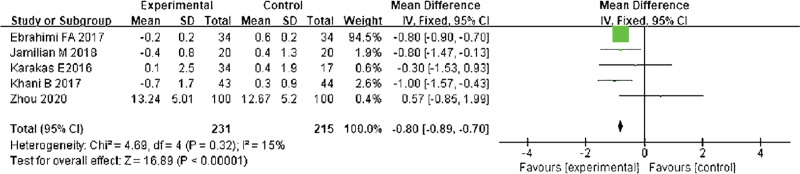
Forest map compared with HOMA-IR. HOMA-IR = HOMA-insulin resistance.

It was possible to perform a meta-analysis of the insulin resistance index HOMA-IR after treatment based on the results of this study. The results of the heterogeneity test indicated that Chi^2^ = 4.69, df = 4, *P* = .32, I^2^ = 15%, indicating that the research data contained in the study showed distinct heterogeneity. The fixed effect model was adopted to analyze (Fig. 7). The study group HOMA-IR decreased after treatment in comparison with the control group (*P* < .05). The heterogeneity test indicated that Chi^2^ = 4.69, df = 4, *P* = .32, I^2^ = 15%. The fixed effect model was adopted (*P* < .05).

#### 3.3.5. Fasting blood glucose (FBS).

**Figure 8.**
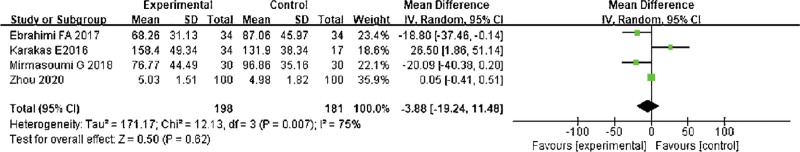
Forest analysis diagram of FBS comparison. FBS = fasting blood glucose.

A meta-analysis was performed on the FBS after treatment. The results of the heterogeneity test indicated that Chi^2^ = 12.13, df = 3, *P* = .007, I^2^ = 75%, indicating that the research data contained in the study showed distinct heterogeneity. The random effect model was adopted to analyze (Fig. 8). There exhibited no remarkable difference in FBS levels after treatment (*P* > .05). This suggests that ω-3 PUFAs had no remarkable effect on FBS in PCOS patients. The heterogeneity test indicated that Chi^2^ = 12.13, df = 3, *P* = .007, I^2^ = 75%. The random effect model was adopted to analyze (*P* > .05).

#### 3.3.6. Low density lipoprotein cholesterol (LDL-C).

**Figure 9.**
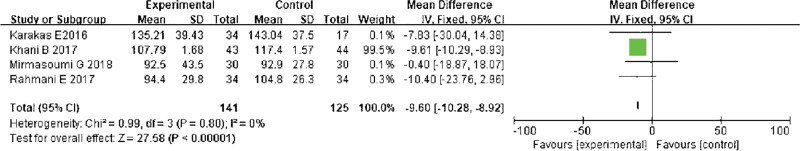
Forest analysis diagram of LDL-C comparison. LDL-C = low density lipoprotein cholesterol.

Meta-analysis of LDL-C after treatment was performed. The results of the heterogeneity test indicated that Chi^2^ = 0.99, df = 3, *P* = .80, I^2^ = 0%, indicating that the research data contained in the study showed distinct heterogeneity, and the fixed effect model analysis shows that (Fig. 9). After treatment, the LDL-C level in the study group was lower (*P* < .05). The heterogeneity test indicated that Chi^2^ = 0.99, df = 3, *P* = .80, I^2^ = 0%. The fixed effect model analysis was used (*P* < .05).

#### 3.3.7. High density lipoprotein cholesterol (HDL-C).

**Figure 10.**
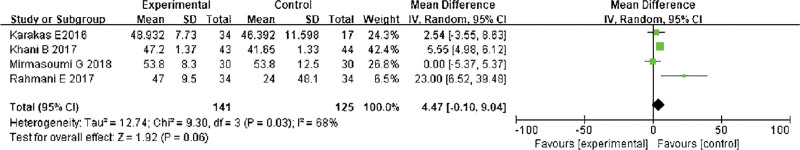
Forest analysis diagram of HDL-C comparison. HDL-C = high density lipoprotein cholesterol.

Meta-analysis of HDL-C after treatment was carried out. The results of the heterogeneity test indicated that Chi^2^ = 11.69, df = 3, *P* = .009, I^2^ = 74%, indicating that the research data contained in the study showed distinct heterogeneity. The random effect model was adopted to analyze (Fig. 10). No remarkable differences were observed in HDL-C levels after treatment (*P* > .05). This indicated that ω-3 PUFAs had no remarkable effect on serum HDL-C in PCOS patients. The heterogeneity test indicated that Chi^2^ = 11.69, df = 3, *P* = .009, I^2^ = 74%. The random effect model was adopted (*P* > .05).

#### 3.3.8. Testosterone (T).

**Figure 11.**
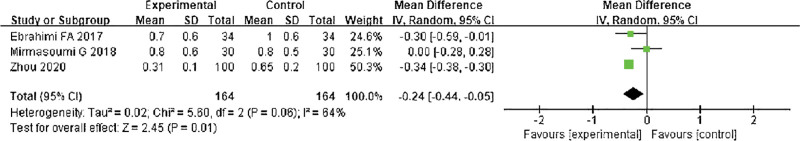
T comparison of forest analysis map.

Meta-analysis was performed on the T levels after treatment. The results of the heterogeneity test indicated that Chi^2^ = 5.60, df = 2, *P* = .06, I^2^ = 64%, indicating that the research data contained in the study showed distinct heterogeneity. The random effect model was adopted to analyze (Fig. 11). Compared with the control group, the T level in the study group was lower after treatment (*P* < .05). The heterogeneity test indicated that Chi^2^ = 5.60, df = 2, *P* = .06, I^2^ = 64%. The random effect model was adopted (*P* < .05).

#### 3.3.9. Ferriman-Galley (mFG).

**Figure 12.**
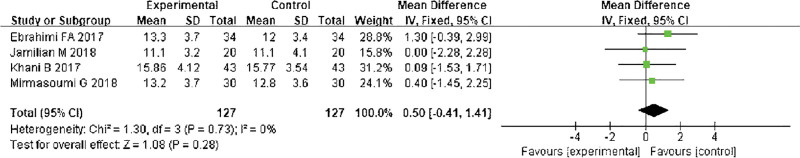
Forest analysis chart with Ferriman-Galley score comparison.

The mFG score after treatment was subjected to meta-analysis. The results of the heterogeneity test indicated that Chi^2^ = 1.30, df = 3, *P* = .73, I^2^ = 0%, indicating that the research data contained in the study showed distinct heterogeneity. The fixed effect model was adopted to analyze (Fig. 12), and there exhibited no remarkable difference in mFG scores (*P* > .05). The heterogeneity test indicated that Chi^2^ = 1.30, df = 3, *P* = .73, I^2^ = 0%. The fixed effect model was adopted to analyze (*P* > .05).

#### 3.3.10. Publication bias analysis.

**Figure 13.**
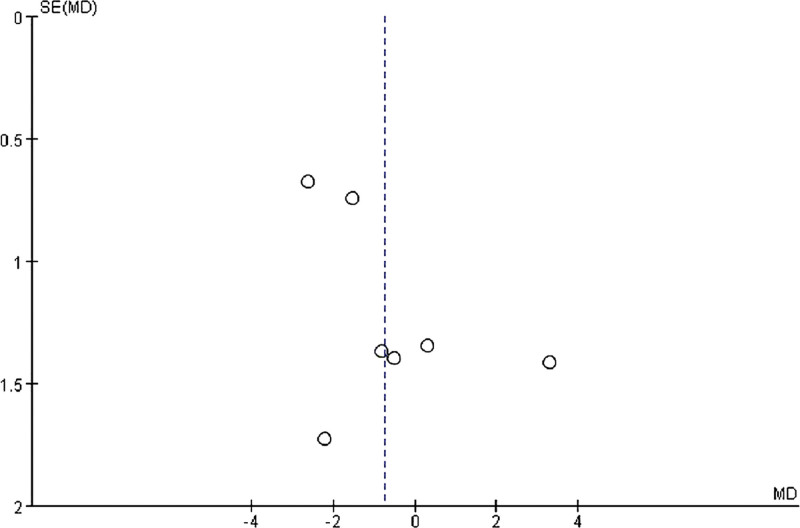
funnel chart based on post-treatment BMI. BMI = body mass index.

The funnel plots constructed with the observed study showed symmetry, and no significant publication bias was detected in funnel plots (Fig. 13). The Egger linear regression test indicated that no significant publication bias was detected in the meta-analyses under different variables (*P* > .05 for all), thus further confirming the robustness of the meta-analysis results.

## 4. Discussion

PCOS is a neuroendocrine and metabolic disease characterized by follicular dysplasia, androgen excess and insulin resistance, with multiple causes and symptoms. In accordance with different criteria, 4% to 8% of women show PCOS, which is the most common cause of irregular menstruation, amenorrhea, and infertility in women of childbearing age. In the meanwhile, PCOS has many negative effects on physiological function and metabolism, such as metabolic syndrome, island element resistance, dyslipidemia, hypertension, hyperinsulinemia.^[23]^ Over the past few years, many studies have shown that oral supplementation of ω-3 PUFAs can remarkably enhance the metabolic and endocrine indicators of PCOS patients.^[24]^ Therefore, this study conducted a meta-analysis of the therapeutic effect of oral supplementation of ω-3 PUFAs on PCOS patients, which is a clinical application of ω-3 PUFAs. ω-3 PUFAs provide evidence-based evidence to treat PCOS patients.

A large proportion of PCOS patients are overweight, obese, or centrally obese. Studies have shown that the prevalence of IGT and diabetes in PCOS patients is correlated with BMI positively.^[25]^ PCOS patients with a BMI > 30 kg/m^2^ had a 4-fold augmented risk of developing from normal glucose tolerance to IGT and diabetes. Obesity has a remarkable impact on the development, progression and even worsening of insulin resistance and hyperinsulinemia. In addition, central obesity is also an important factor in insulin resistance. These PCOS patients with more abdominal fat also have a higher risk of developing insulin resistance. The BMI of the study group had no remarkable difference with control group, which indicated that ω-3 PUFAs had a remarkable effect on the BMI value of PCOS patients. There is no apparent effect and no remarkable increasing or decreasing of the patient BMI.

Most patients with PCOS have abnormal metabolic function, combined with systemic or abdominal obesity, lipid metabolism disorder, and insulin resistance. In this condition, TG, TC, and LDL-C increase, while HDL-C decreases. Many previous studies have indicated that ω-3 fatty acids can enhance hypertriglyceridemia, inflammatory response, platelet aggregation, arrhythmia and atherosclerosis. In addition, related studies have indicated that ω-3 fatty acids can enhance cardiovascular disease.^[26]^ Regardless of cardiovascular disease, intake of fish or ω-3 fatty acids can remarkably reduce the mortality of coronary atherosclerotic heart disease (including fatal myocardial infarction and sudden cardiac death).^[27]^ The meta-analysis of the levels of TC, TG, and LDL-C in the study group following treatment were largely declined (*P* < .05). However, there was no remarkable difference in HDL-C levels, indicating that ω-3 PUFAs have an important and positive role in improving fat metabolism disorders in PCOS patients and preventing long-term cardiovascular and cerebrovascular-related complications. Patients with PCOS have a much lower risk of cardiovascular disease with improved lipid levels, which can also be of great benefit to PCOS.

The International Diabetes Federation lists PCOS as one of the risk factors closely related to diabetes.^[28]^ Patients with PCOS are prone to acquiring diabetes mellitus. Obesity and insulin resistance are the main factors; in addition, age, family history of diabetes, high androgen levels, history of gestational diabetes, high waist-to-hip ratio and high TG can also increase the risk of diabetes to some extent in patients with PCOS. Therefore, early intervention in PCOS patients can reduce the risk of impaired FBS, IGT and even diabetes. There exhibited no remarkable difference in the level of FBS after treatment. This indicated that ω-3 PUFAs had no remarkable effect on the FBS of PCOS patients, and the FBS level of patients would not fluctuate remarkably after medication, which will not increase the risk of diabetes.

Insulin resistance is a key factor in the occurrence and development of diabetes, and insulin resistance is widely present in PCOS patients.^[29]^ IGT and diabetes mellitus are also more likely to occur when insulin resistance is severe. Some scholars have believed that insulin resistance and compensatory hyperinsulinemia may enhance the body androgen activity, and high levels of androgens may further aggravate insulin resistance by changing the body metabolism, thus forming a vicious circle.^[30,31]^ A recent systematic review indicated that insulin sensitivity decreased remarkably in PCOS patients. In addition to the effects of BMI and age factors, insulin sensitivity was reduced by 27% in patients with PCOS. In addition, the increase of BMI can aggravate the insulin resistance in patients with PCOS. According to the meta-analysis of HOMA-IR, the HOMA-IR of the study group was lower, which indicated that ω-3 PUFAs could effectively improve the insulin resistance of PCOS patients. After the HOMA-IR level of patients decreased, the risk of diabetes decreased, which would form a virtuous circle and help to improve their prognosis.

Hyperandrogenemia is a specific change in patients with PCOS, and its pathogenesis is very complex.^[32,33]^ The main clinical symptoms are hirsutism, acne and alopecia. In severe cases, it will cause menstrual disorders and ovulation disorders, leading to infertility. T is an effective index to evaluate ovarian androgen. Several studies have suggested that the intake of ω-3 PUFAs has a certain intervention effect on the complications caused by high androgen levels in PCOS patients, such as hirsutism and hair loss caused by sebaceous hyperplasia. Meta-analysis of TC levels and post-Ferriman-Galley scores in the study group was lower after treatment, but the 2 groups. There exhibited no remarkable difference in Ferriman-Galley score after treatment. It is suggested that supplementation of ω-3 PUFAs can only effectively reduce the T level of patients but has no remarkable effect on improving Ferriman-Galley score. The mechanism is that ω-3 PUFAs can inhibit the expression of steroid hormone synthesis acute regulatory protein gene by inhibiting arachidonic acid, which then regulates the hormone level in vivo.^[34]^ The limitations were that the inclusion and exclusion criteria were relatively strict, with the final small number of literatures. The clear standards for dose and duration of supplementation need further study. In addition, clinical trials of combined supplementation with other nutrients should be considered in the future.

With 7 clinical controlled studies comprising 574 samples, our study provides a robust dataset for the analysis. Unlike many meta-analyses, we have undertaken a meticulous evaluation of the methodology quality in the included studies, accounting for various risk factors and biases. We evaluated not only commonly assessed markers such as BMI and lipid profile but also other markers of clinical significance in PCOS like insulin resistance (HOMA-IR), T levels, and Ferriman-Galley scores. The meta-analysis employed both fixed and random-effect models where appropriate, accounting for heterogeneity among studies, thus providing a more reliable set of conclusions. Our study also checked for publication bias and found none, further corroborating the robustness of our meta-analysis results. While ω-3 PUFAs did not show a significant impact on BMI or fasting blood sugar levels, our study revealed that they have a beneficial effect on total cholesterol levels, TG, HOMA-IR, and T levels. This suggests that while ω-3 PUFAs may not address all aspects of PCOS, they could play an important role in managing some of its biochemical manifestations.

Mechanistically, ω-3 PUFAs exert their beneficial effects in PCOS through multiple pathways. They modulate the activity of peroxisome proliferator-activated receptors (PPARs), thereby influencing lipid metabolism and insulin sensitivity.^[35]^ Additionally, ω-3 PUFAs have anti-inflammatory properties that reduce systemic inflammation, a key contributor to endocrine dysregulation in PCOS.^[36,37]^ These combined actions contribute to improved metabolic and hormonal profiles in PCOS patients. In this meta-analysis, we scrutinized the effect of ω-3 PUFAs on various biochemical markers and clinical indices in patients with PCOS. The comprehensive literature search, rigorous inclusion criteria, and quality assessment make this meta-analysis a substantial addition to the existing literature.

Our meta-analysis showed beneficial effects of ω-3 fatty acids on cholesterol, TG, and insulin resistance in PCOS patients. These effects align with established mechanisms: Omega-3s lower hepatic TG production, act as anti-inflammatory agents, enhance insulin sensitivity, and inhibit androgen biosynthesis. Our findings underscore the therapeutic potential of ω-3 fatty acids in managing metabolic markers in PCOS.

One limitation of our study should be noted. As pointed out by the reviewer, 3 of the included studies used a combination of omega-3 and vitamin supplements, rather than omega-3 alone. While we chose not to omit these studies due to the limited availability of literature, this combination therapy could confound the results related to the efficacy of omega-3 fatty acids in isolation. We acknowledge that this aspect may impact the specificity of our findings and concur that further research involving omega-3 supplementation alone is warranted for a more thorough evaluation of its impact on PCOS management.

## 5. Conclusion

To sum up, oral supplementation of omega-3 PUFAs has a certain clinical effect on PCOS patients, which can improve their endocrine levels and reduce blood lipid levels. This treatment is feasible in clinical treatment, especially for PCOS patients with insulin resistance or/ and high TC (especially LDL-C) and TG. The treatment time of all studies is not consistent, so there are some limitations, which need to be followed up by scholars to provide more support for the clinical application of ω-3 PUFAs in patients with PCOS.

## Author contributions

**Data curation:** Yue Huang.

**Formal analysis:** Yue Huang, Xiang Zhang.

**Investigation:** Xiang Zhang.

**Methodology:** Yue Huang.

**Resources:** Xiang Zhang.

**Writing – original draft:** Yue Huang, Xiang Zhang.

**Writing – review & editing:** Yue Huang.
